# Prevalence of Circadian Rhythm Sleep-Wake Disorder in Outpatients with Schizophrenia and Its Association with Psychopathological Characteristics and Psychosocial Functioning

**DOI:** 10.3390/jcm10071513

**Published:** 2021-04-05

**Authors:** Kentaro Matsui, Ken Inada, Kenichi Kuriyama, Takuya Yoshiike, Kentaro Nagao, Hidehiro Oshibuchi, Rie Akaho, Katsuji Nishimura

**Affiliations:** 1 Department of Psychiatry, Tokyo Women’s Medical University, Tokyo 1628666, Japan; inada.ken@twmu.ac.jp (K.I.); oshibuchi.hiderhiro@twmu.ac.jp (H.O.); akaho.rie@twmu.ac.jp (R.A.); nishimura.katsuji@twmu.ac.jp (K.N.); 2 Clinical Laboratory, National Institute of Mental Health, National Center of Neurology and Psychiatry, Tokyo 1878551, Japan; 3 Department of Sleep-Wake Disorders, National Institute of Mental Health, National Center of Neurology and Psychiatry, Tokyo 1878551, Japan; kenichik@ncnp.go.jp (K.K.); yoshiike@ncnp.go.jp (T.Y.); knagao@ncnp.go.jp (K.N.)

**Keywords:** schizophrenia, circadian rhythm, sleep-wake cycle, social dysfunction, anxiety

## Abstract

The prevalence of circadian rhythm sleep-wake disorder (CRSWD) among patients with schizophrenia is not clear. The effect of comorbid CRSWD on such patients has also not been fully evaluated yet. Outpatients with schizophrenia in the maintenance phase who visited Tokyo Women’s Medical University Hospital between April 2018 and March 2019 participated in this study. The Brief Psychiatric Rating Scale (BPRS), the Clinical Global Impressions–Severity Illness Scale (CGI-S), Global Assessment of Functioning (GAF), World Health Organization Disability Assessment Schedule II, Insomnia Severity Index (ISI), and Morningness–Eveningness Questionnaire (MEQ) were administered, and the patient responses with and without CRSWD were compared. Of the 105 patients with schizophrenia, 19 (18.1%) had CRSWD. There were trends toward higher BPRS and lower GAF scores in the CRSWD group than in the non-CRSWD group, although these did not reach statistical significance following a false discovery rate correction. Among the BPRS subitems, the anxiety scores were significantly higher in the CRSWD group than in the non-CRSWD group (*p* < 0.01). CRSWD was highly prevalent among patients with schizophrenia in the maintenance phase. Comorbidities of CRSWD may affect psychopathological characteristics and psychosocial functioning.

## 1. Introduction

Schizophrenia is a chronic mental disorder with a lifetime prevalence of 0.72% [1]. Positive symptoms, such as auditory hallucinations and paranoia, and negative symptoms, such as lack of motivation and interest, flat affect, and social withdrawal, as well as cognitive impairment, are major burdens on patients’ lives [2]. The incidence of insomnia is high, particularly in the acute phase [3], and it may persist even after other acute-phase symptoms improve with the use of antipsychotics [4,5,6]. Negative symptoms can also persist after the improvement of acute-phase symptoms, resulting in social dysfunction [7,8], which may further affect circadian rhythms as a result of reduced daytime activity. Moreover, antipsychotics that are usually used in the treatment of schizophrenia can attenuate the diurnal variation in sleep-wake behavior through a sedative effect that can persist during the day [9]. Although the impact of the psychopathological characteristics and sedative properties of antipsychotics are not clear, the disruption of sleep-wake rhythms has been reported in some patients with schizophrenia [10,11,12,13]. However, so far, few studies on sleep and circadian rhythms in patients with schizophrenia have focused on circadian rhythm sleep-wake disorder (CRSWD) in the International Classification of Sleep Disorders—Third Edition (ICSD-3) [14,15,16,17].

Indeed, no study on the prevalence of CRSWD among patients with schizophrenia has yet been conducted. Furthermore, the negative effect of CRSWD on the psychosocial functioning of patients with schizophrenia is also unclear. In a recent case-control study, researchers compared the symptoms of patients with schizophrenia with and without delayed sleep-wake phase disorder (DSWPD), which is defined by a sleep period that is abnormally late in comparison with conventional or socially desirable times [14]. In this study, patients with DSWPD had significantly worse negative symptoms and social functioning than those without DSWPD [18]. The case of a patient with schizophrenia where social interaction and communication increased after the improvement in the circadian rhythms [19] also suggests that stabilization of the sleep-wake rhythm may improve social dysfunction. However, the relationship between disrupted sleep-wake rhythms and psychosocial functioning is still unclear, with scarce information about the prevalence rates in a clinical population of patients with schizophrenia [10,19]. Therefore, we hypothesized that the comorbidity of sleep disturbance may have a negative impact on patients with schizophrenia, especially those in the maintenance phase who are on the road to recovery. We therefore conducted a cross-sectional observational study to clarify the prevalence of CRSWD among outpatients with schizophrenia in the maintenance phase and investigate the effect of CRSWD on patients’ psychopathological characteristics and psychosocial functioning.

## 2. Materials and Methods

### 2.1. Study Population and Outcomes

This study was conducted in accordance with the Declaration of Helsinki. The protocol of this study was reviewed and approved by the ethics committee of the Tokyo Women’s Medical University (approval code: 4660). All patients provided written informed consent to participate after being given a thorough explanation of the study. Fully trained attending psychiatrists had diagnosed schizophrenia according to the Diagnostic and Statistical Manual of Mental Disorders, Fifth Edition [2], and the patients had regularly visited the Neuropsychiatric Department in Tokyo Women’s Medical University Hospital for years. The psychiatrists also documented patients’ living conditions, clinical history (including hospitalization), and comorbidities of other diseases on electronic medical charts during routine clinical interviews. We recruited consecutive patients with schizophrenia aged ≥20 years who visited our hospital between April 2018 and March 2019. Subjects were excluded if psychiatrists noted any of the following during clinical examination: (a) occurrence of acute psychotic states during the previous year, (b) psychiatric hospitalization in the previous three months, (c) employment as a shift worker, (d) presence of obstructive sleep apnea and restless legs syndrome (screened for habitual snoring and leg discomfort that worsens in the evening, respectively), (e) ongoing alcohol or substance abuse, and (f) suicide risk.

Demographic information (age, sex, height, and weight), comorbidities, duration of schizophrenia, and use of prescribed medications were confirmed through a medical record review. Daily doses of antipsychotics and benzodiazepine receptor agonists (including Z-drugs) were changed to chlorpromazine and diazepam daily dose equivalents, respectively [20]. The Brief Psychiatric Rating Scale (BPRS) [21] and Clinical Global Impressions–Severity of Illness Scale (CGI-S) [22] were used to assess the severity of psychiatric symptoms. The Global Assessment of Functioning (GAF) [23] was used to measure psychosocial functioning. In addition, the participants were asked to answer questionnaires, including the 12-item Self-Report World Health Organization Disability Assessment Schedule II (WHO-DAS II) [24] for measuring functional impairment, the Insomnia Severity Index (ISI) for assessing the insomnia severity [25,26], and the Morningness–Eveningness Questionnaire (MEQ) for evaluating the chronotype [27,28]. The questionnaires also included items about their lifestyle and habits, such as habitual drinking and smoking, employment, and participation in psychiatric rehabilitation programs. CRSWD was diagnosed in accordance with the ICSD-3 [14] by a board-certified sleep specialist and was based on a clinical interview regarding their sleep problems and a sleep log completed by patients for at least two weeks (preferably four weeks). Participants who met the CRSWD criteria were further assigned to the following subcategories: DSWPD, advanced sleep-wake phase disorder, irregular sleep-wake rhythm disorder (ISWRD), and non-24-h sleep-wake disorder [14].

### 2.2. Statistical Analysis

The Mann–Whitney *U* test was used to compare continuous variables and scores on ISI, MEQ, BPRS (total, positive subscale, and negative subscale), CGI-S, GAF, and WHO-DAS II between the participants who met the criteria for CRSWD (CRSWD group) and those who did not meet the criteria for CRSWD (non-CRSWD group). The chi-squared test was used to compare the categorical variables between the CRSWD and non-CRSWD groups. The Mann–Whitney *U* test was also used to compare the BPRS subitems between the two groups. Benjamini–Hochberg false discovery rate (FDR) corrections [29] were performed to minimize the likelihood of Type I errors due to multiple comparisons. SPSS software (version 26.0; SPSS Japan, Inc., Tokyo, Japan) was used to conduct all analyses. FDR corrected *p* < 0.05 was considered significant, and uncorrected *p* < 0.05 was regarded as showing a trend.

## 3. Results

### 3.1. Comorbidity of CRSWD

Of the 139 patients with schizophrenia, 105 were included in the analysis; 31 patients who did not complete a sleep log for >2 weeks and three patients who withdrew their consent were excluded. Of the 105 patients, 19 (18.1%; 95% confidence interval [CI], 10.7 to 25.5) met the criteria for CRSWD in ICSD-3; these patients included 13 (12.4%; 95% CI, 6.1 to 18.7) with DSWPD, five (4.8%; 95% CI, 0.7 to 8.8) with ISWRD, and one (1.0%; 95% CI, −0.9 to 2.8) with advanced sleep-wake phase disorder. Nobody in this cohort had non-24-h sleep-wake disorder. All patients were treated with antipsychotics.

### 3.2. Descriptive and Clinical Variables

Compared with the non-CRSWD group, the CRSWD group had a significantly higher ISI score (*p* < 0.001, with an effect size of 0.34 and power of 0.264) and a significantly lower MEQ score (*p* < 0.001, with an effect size of 0.34 and power of 0.264). The CRSWD group tended to be younger (*p* < 0.01), have a higher BMI (*p* < 0.01), have lower rates of employment and regular participation in outpatient rehabilitation programs (*p* < 0.05), and be more likely to be suvorexant users (*p* < 0.05) compared with the non-CRSWD group; however, these were not significant following FDR correction. The dosages of antipsychotics or benzodiazepine receptor agonists and other descriptive variables were comparable between the two groups (Table 1).

### 3.3. Psychopathological Characteristics and Psychosocial Functioning

The BPRS total score for general psychopathology tended to be higher in the CRSWD group than in the non-CRSWD group (*p* < 0.05); however, this was not significant following FDR correction. The BPRS positive and negative subscales and CGI-S scores were comparable between the two groups. For psychosocial functioning, the GAF score tended to be higher in the CRSWD group than in the non-CRSWD group (*p* < 0.05); however, this was not significant following FDR correction. The WHO-DAS II scores were comparable between the two groups (Table 2). Among the BPRS subitems, the score for anxiety was significantly higher in the CRSWD group than in the non-CRSWD group (*p* < 0.01, with an effect size of 0.33 and power of 0.251), which remained significant following FDR correction. The score for somatic concern tended to be higher in the CRSWD group than in the non-CRSWD group (*p* < 0.05); however, this was not significant following FDR correction. Other BPRS subitem scores were comparable between the two groups (Table 3).

## 4. Discussion

To our knowledge, this is the first study on the prevalence of CRSWD among patients with schizophrenia. In this study, 18.1% of outpatients with schizophrenia in the maintenance phase had comorbid CRSWD. The comorbidity rate was similar to those of CRSWD and DSWPD in the maintenance phase of bipolar disorder (32.4% and 10.4–26.0%, respectively) [30,31] and to that of CRSWD in the maintenance phase of major depressive disorder (9.6%) [32]. These rates seem to be higher than those in the general population (<1–5.3%) [33,34,35], which suggests that psychopathological processes underlying these disorders could be factors for vulnerability to CRSWD. In recent years, there has been increasing evidence of the immune control of circadian rhythms by regulating the transcription of clock genes [36]. Inflammatory cytokines, which have been reported to be associated with poor sleep in both mood disorders and schizophrenia [37], may be associated with the comorbidity of these disorders with CRSWD.

In addition, in this study the psychopathological manifestations had a tendency to be more severe in the CRSWD group than in the non-CRSWD group. A previous study of patients with schizophrenia implied that sleep-wake rhythms were more disrupted in patients with predominantly positive symptoms than in those with predominantly negative symptoms [10]. Another study implied that patients with DSWPD had higher total and negative scores on the Positive and Negative Syndrome Scale than those without DSWPD [18]. However, it should be noted that the sample sizes of both studies were small. Scores for the BPRS subitems suggested that anxiety was significantly associated with CRSWD in this study. CRSWD has been reportedly associated with pathological underpinnings of anxiety [38,39], and this may also be true for patients with schizophrenia. In particular, DSWPD may develop from hyperarousal before sleep onset due to anxiety or psychological stress. The effects of anxiety can play a role in the prognosis of patients with schizophrenia in terms of both daytime functioning and quality of life [40]. Moreover, anxiety has been considered to be related to serotonergic or noradrenergic dysfunction, rather than to dopamine dysfunction, which underlies the pathological processes of schizophrenia [41,42]. In view of this possibility, future research on CRSWD in schizophrenia should explore therapeutic interventions different to conventional pharmacotherapy for psychotic symptoms.

Studies on circadian rhythms in schizophrenia suggest irregularities in sleep-wake rhythms [11,12,13,43]. ISWRD was found in some patients in this study, which is consistent with the results of previous reports. In contrast, the finding that DSWPD was more common than ISWRD was different from the results of previous studies; a daily circadian rhythm cycle of 23.7 h [44], an advance in melatonin secretion rhythms [45], and an equal number of patients with ISWRD and advanced sleep-wake phase disorder but no patients with DSWPD [46] have been reported, suggesting a trend toward a disruption, but not a delay, of circadian rhythms in patients with schizophrenia. However, the results of this study suggest that a certain number of outpatients with schizophrenia have delayed sleep-wake rhythms in the clinical setting, which is consistent with a report by Wulff et al. [11]. Furthermore, a trend toward a higher use of suvorexant by patients with CRSWD than by those without CRSWD could be a consequence of treatment for difficulty falling asleep or increased wakefulness after sleep onset as an endophenotype of the disrupted sleep-wake rhythm [47]. Taken together, part of the insomnia symptoms in chronic schizophrenia [4,5,6] may be related to CRSWD, especially DSWPD, which accounted for approximately 70% of cases of CRSWD in this study. Of the patients with schizophrenia in this study, those in the CRSWD group tended to be younger than those in the non-CRSWD group; this is consistent with the reports of relatively higher rates of DSWPD among adolescents (1.1–15.9%) [39,48,49,50,51]. Despite these implications, the diagnosis of CRSWD in this study was based on patients’ behavioral features in clinical settings; hence, it is possible that objective measures, such as melatonin secretion onset and diurnal variation in the core body temperature, may deviate from the subcategories of CRSWD. Comparing self-reported sleep schedules, sleep log recordings, and actigraphy may also contribute to discrepancies. These possibilities need to be examined in future studies.

Another finding in this study is that comorbid CRSWD may be associated with psychosocial functioning. One study demonstrated no correlation between circadian rhythm and social dysfunction in patients with schizophrenia, but it focused on older adult patients and included both inpatients and outpatients [52]. To the best of our knowledge, our study is the first to focus on the relationship between CRSWD and psychosocial functioning in outpatients with schizophrenia across a wide age range. This study implied that CRSWD could be related not only to scores of psychosocial functioning but also of the ability to work. Because participation in employment and rehabilitation programs can be social zeitgebers, it is unclear whether participation in social activities affects circadian rhythm disruptions or whether circadian rhythm disruptions prevent participation in social activities. Although the causal relationship remains unclear, therapeutic interventions with add-on melatonin agonists, which have been shown to be effective for better sleep-wake behavior and the alleviation of psychopathological symptoms in patients with schizophrenia [53,54,55], may enable patients to participate in social activities.

This study had several limitations. First, it focused only on the prevalence of CRSWD in a small sample of outpatients at a single center, and the results may not be representative of patients with schizophrenia in general. Second, the weakness of this study is the low statistical power due to the small number of subjects, in the CRSWD group especially. Even though the ISI score, MEQ score, and anxiety (a subitem of BPRS) were significantly different between the CRSWD and non-CRSWD groups even following FDR correction, the effect sizes were small and the statistical power was low. Moreover, the invariance of the measurements used in the study, especially in the CRSWD group, was not verified. Third, the timing of the onset of CRSWD was not assessed. CRSWD was diagnosed on the basis of a dysregulation of the sleep-wake rhythm that had lasted at least three months, as indicated in the diagnostic criteria [14], but whether it was a temporary or permanent change was not examined in this study. Moreover, the diagnosis was not based on objective measures, such as the melatonin level, deep body temperature, and actigraphy. Therefore, sleep misperceptions may not have been adequately assessed. Although we screened for habitual snoring and leg discomfort, obstructive sleep apnea and restless legs syndrome were not confirmed by polysomnography, which is another limitation of this study. Fourth, CRSWD in this study included DSWPD, ISWRD, and advanced sleep-wake phase disorder. This heterogeneity may have influenced the results. Among them, DSWPD and ISWRD were suggested to have relatively high degrees of comorbidity with schizophrenia; thus, future studies should focus on the characteristics of each when they are comorbid. Fifth, cognitive impairment was not assessed in this study. The effect of cognitive impairment, especially with regard to social dysfunction [56], cannot be ignored. Sixth, the data did not address the duration of medication use. The duration of psychotropic medication may affect CRSWD development through sedation, and this possibility should be examined in future studies.

## 5. Conclusions

CRSWD was observed in approximately 18% of our sample of outpatients with schizophrenia who were in the maintenance phase. CRSWD can be an important comorbidity that exacerbates social dysfunction in schizophrenia. Furthermore, anxiety, rather than schizophrenia-specific positive and negative symptoms, may be associated with CRSWD. Future research should examine the comorbid burden of schizophrenia and CRSWD, including the effects of anxiety and cognitive impairment. The findings of this study were obtained from a small sample. Therefore, replication is required in a larger-scale study.

## Figures and Tables

**Table 1:** Descriptive statistics for the entire study population.

Characteristic	Total Population (*n* = 105)	CRSWD Group (*n* = 19)	Non-CRSWD Group (*n* = 86)	*p* ^1^
Age (years) at time of investigation, mean (SD)	47.5 (12.6)	41.2 (13.2)	48.9 (12.0)	0.009
Female, *n*	60 (57.1%)	12 (63.2%)	48 (55.8%)	0.558
Body mass index (kg/m^2^), mean (SD)	24.2 (4.2)	26.6 (4.5)	23.7 (4.0)	0.009
Length (years) of disease morbidity, mean (SD)	18.8 (10.9)	15.9 (9.2)	19.5 (11.2)	0.172
Habitual alcohol ingestion, *n*	17 (16.2%)	3 (15.8%)	14 (16.3%)	0.958
Habitual smoking, *n*	16 (15.2%)	2 (10.5%)	14 (16.3%)	0.528
Work or rehabilitation engagement				
Employment or regular participation in outpatient rehabilitation programs, *n*	38 (36.2%)	3 (15.8%)	35 (40.7%)	0.041
Working > 15 h per week, *n*	17 (16.2%)	1 (5.3%)	16 (18.6%)	0.153
Working > 30 h per week, *n*	10 (9.5%)	1 (5.3%)	9 (10.5%)	0.485
Concomitant medications				
Mood stabilizers, ^2^ *n*	24 (22.9%)	7 (36.8%)	17 (19.8%)	0.109
Ramelteon, *n*	2 (1.9%)	1 (5.3%)	1 (1.2%)	0.237
Suvorexant, *n*	9 (8.6%)	4 (21.4%)	5 (5.8%)	0.032
BZRAs, *n*	65 (61.9%)	13 (68.4%)	52 (60.5%)	0.518
Dose (mg) of antipsychotics, ^3^ mean (SD)	532.7 (410.0)	453.2 (241.0)	550.3 (437.7)	0.851
Dose (mg) of BZRAs, ^4^ mean (SD)	8.5 (10.5)	8.1 (10.2)	8.5 (10.6)	0.979
ISI score (points), mean (SD)	7.4 (5.9)	11.8 (5.8)	6.4 (5.6)	<0.001 *
MEQ score (points), mean (SD)	53.9 (9.8)	46.0 (11.6)	55.6 (8.5)	<0.001 *

^1^ Chi-square test (categorical variables) or Mann–Whitney *U* test (continuous variables). ^2^ Including lithium, valproate, carbamazepine, and lamotrigine. ^3^ Chlorpromazine equivalent. ^4^ Diazepam equivalent. * *p* values significant following FDR corrections; CRSWD, circadian rhythm sleep-wake disorder; SD, standard deviation; BZRA, benzodiazepine receptor agonist; ISI, Insomnia Severity Index; MEQ, Morningness–Eveningness Questionnaire; FDR, False Discovery Rate.

**Table 2:** Comparison of psychopathological characteristics and psychosocial functioning.

Assessment	Total Population (*n* = 105)	CRSWD Group (*n* = 19)	Non-CRSWD Group (*n* = 86)	*p* ^1^
BPRS total score (points)	29.0 (7.7)	32.0 (7.5)	28.3 (7.6)	0.039
BPRS positive subscale score (points)	6.9 (3.4)	7.7 (3.7)	6.7 (3.3)	0.263
BPRS negative subscale score (points)	6.2 (2.5)	6.3 (2.0)	6.2 (2.6)	0.605
CGI-S score (points)	3.5 (1.0)	3.7 (1.0)	3.4 (1.0)	0.362
GAF score (points)	56.7 (11.2)	51.1 (9.2)	57.9 (11.3)	0.033
WHO-DAS II score (points)	22.2 (8.4)	24.7 (9.0)	21.7 (8.2)	0.116

All data are presented as means (standard deviations). No values were significant following FDR corrections. ^1^ Mann–Whitney *U* test. CRSWD, circadian rhythm sleep-wake disorder; SD, standard deviation; BPRS, Brief Psychiatric Rating Scale; CGI-S, Clinical Global Impressions–Severity of Illness; GAF, Global Assessment of Functioning; WHO-DAS II, World Health Organization Disability Assessment Schedule II.

**Table 3:** Scores for BPRS subitems.

BPRS Subitems	CRSWD (*n* = 19)	Non-CRSWD (*n* = 86)	*p* ^1^
Somatic concern	2.21 (1.27)	1.74 (1.13)	0.046
Anxiety	3.21 (1.32)	2.07 (1.13)	0.001 *
Emotional withdrawal	1.63 (0.60)	1.77 (0.81)	0.644
Conceptual disorganization	1.84 (0.90)	1.92 (0.97)	0.776
Guilt	2.00 (1.29)	1.52 (0.85)	0.097
Tension	1.37 (0.68)	1.17 (0.41)	0.244
Bizarre behavior	1.11 (0.46)	1.35 (0.72)	0.077
Grandiosity	1.26 (0.73)	1.24 (0.70)	0.853
Depressed mood	1.90 (1.10)	1.61 (0.86)	0.339
Hostility	1.00 (0.00)	1.06 (0.36)	0.411
Suspiciousness	2.21 (1.47)	1.86 (1.36)	0.237
Hallucinations	2.68 (1.95)	1.95 (1.48)	0.103
Motor retardation	2.90 (1.29)	2.55 (1.38)	0.263
Uncooperativeness	1.05 (0.23)	1.01 (0.11)	0.239
Unusual thought content	1.90 (1.49)	1.63 (1.36)	0.314
Blunted affect	1.74 (0.65)	1.84 (0.88)	0.875
Excitement	1.00 (0.00)	1.05 (0.26)	0.411
Disorientation	1.00 (0.00)	1.01 (0.11)	0.638

All data are presented as means (standard deviations). ^1^ Mann–Whitney *U* test; * *p* values significant following FDR corrections. BPRS, Brief Psychiatric Rating Scale; CRSWD, circadian rhythm sleep-wake disorder.

## Data Availability

The data presented in this study are available from the corresponding author upon reasonable request.
